# Aerobic exercise alleviates pyroptosis-related diseases by regulating NLRP3 inflammasome

**DOI:** 10.3389/fphys.2022.965366

**Published:** 2022-09-15

**Authors:** Shujuan Hu, Xingxia Wan, Xianhui Li, Xianwang Wang

**Affiliations:** ^1^ School of Education and Physical Education, Yangtze University, Jingzhou, China; ^2^ School of Physical Education and Science, Jishou University, Jishou, China; ^3^ Department of Biochemistry and Molecular Biology, Health Science Center, Yangtze University, Jingzhou, China; ^4^ College of Pharmacy, Jishou University, Jishou, China

**Keywords:** aerobic exercise, pyroptosis, pyroptosis-related diseases, mechanisms, NLRP3 inflammasome

## Abstract

Pyroptosis plays a crucial role in a variety of human diseases, including atherosclerosis, obesity, diabetes, depression, and Alzheimer’s disease, which usually release pyroptosis-related cytokines due to inflammation. Many studies have demonstrated that aerobic exercise is a good option for decreasing the release of pyroptosis-related cytokines. However, the molecular mechanisms of aerobic exercise on pyroptosis-related diseases remain unknown. In this review, the effects of aerobic exercise on pyroptosis in endothelial cells, adipocytes and hippocampal cells, and their potential mechanisms are summarized. In endothelial cells, aerobic exercise could inhibit NOD-like receptor protein 3 (NLRP3) inflammasome-mediated pyroptosis by improving the endothelial function, while reducing vascular inflammation and oxidative stress. In adipocytes, aerobic exercise has been shown to inhibit pyroptosis by ameliorating inflammation and insulin resistance. Moreover, aerobic exercise could restrict pyroptosis by attenuating microglial activation, neuroinflammation, and amyloid-beta deposition in hippocampal cells. In summary, aerobic exercise alleviates the pyroptosis-related diseases by regulating the NLRP3 inflammation si0067naling.

## Introduction

**FIGURE 1:**
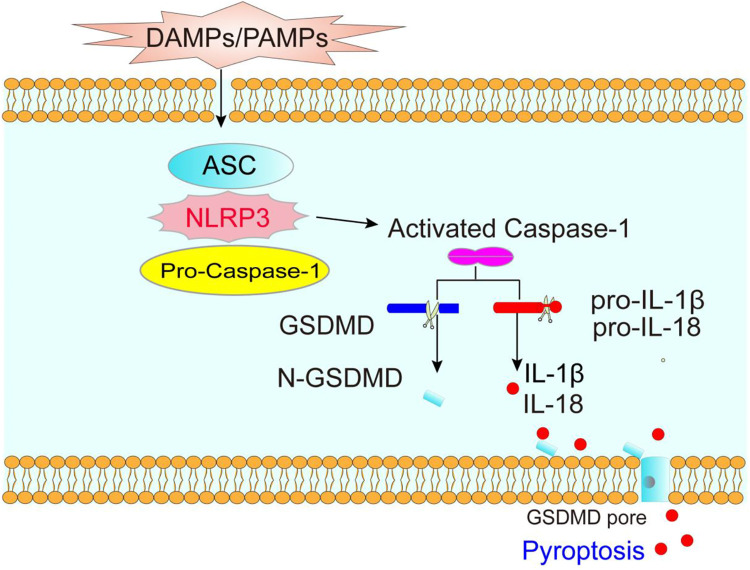
The molecular mechanism of pyroptosis. DAMPs (danger-associated molecular patterns) and PAMPs (pathogen-related molecular patterns) activate NLRP3 inflammasome, promotes Caspase-1 activation, which cleavages GSDMD and the precursor of IL-1β and IL-18, forming mature IL-1β and IL-18, thereby causing pyroptosis.

Pyroptosis, a type of lytic programmed cell death caused by inflammasomes, is an important natural immune response in our body (Kovacs and Miao 2017). Pore formation in the plasma membrane, swelling and rupture of cells, massive leakage of cytoplasmic contents, and release of inflammatory factors are typical features of pyroptosis (Man et al., 2017). Pyroptosis is induced by the NOD-like receptor protein 3 (NLRP3) inflammasome, and triggered by Caspase-1 (Tang et al., 2020), which controls the N-terminal domain of gasdermin D (GSDMD) by assembling channels in the cell membrane and activates interleukin (IL)-1β and IL-18 (Schroder and Tschopp 2010) (Figure 1).

Pyroptosis occurs in multiple cell types (Shi et al., 2017), including endothelial cells, adipocytes and hippocampal cells. Many studies have suggested that pyroptosis takes an important role in the development of human diseases, including obesity (Mardare et al., 2016), diabetes (Vandanmagsar et al., 2011), atherosclerosis (Hong et al., 2021), Alzheimer’s disease (AD) (Liang et al., 2020), and depression (Liu et al., 2015). Aerobic exercise exhibits an obvious anti-inflammatory effect and is closely related to pyroptosis (Kar et al., 2019). As it is known, aerobic exercise could reduce chronic inflammation and effectively inhibit the expression of inflammatory factors, thereby increasing the release of anti-inflammatory cytokines. Previous studies have found that aerobic exercise could decrease the expression of NLRP3 inflammasome and markedly inhibit the activation of ASC, Caspase-1, IL-1β, and IL-18 (Kar et al., 2019; Lee et al., 2020). Although aerobic exercise can regulate cell pyroptosis, its specific effects on pyroptosis-related diseases and potential mechanisms still need further clarification.

The present review aimed to identify the relationship between aerobic exercise and NLRP3 inflammasome-mediated pyroptosis in endothelial cells, adipocytes, and hippocampal cells, and to investigate the potential mechanism of the effect of aerobic exercise on pyroptosis-related diseases.

## Aerobic exercise and endothelial cell pyroptosis-related diseases

Endothelial cell’s pyroptosis is among the major causes of cardiovascular diseases (Zhang L. et al., 2019). Aerobic exercise is an important strategy to control the endothelial cell’s pyroptosis, and inhibiting the development of cardiovascular diseases.

### Endothelial cell’s pyroptosis and its related diseases

Endothelial cells are considered to be an important modulator in vascular homeostasis, regulated by various paracrine factors, and they play a critical role in maintaining normal vascular tension and blood flow and in inhibiting vascular inflammation and oxidative stress. Endothelial dysfunction is a classical symbol and predictor of cardiovascular diseases (Bai et al., 2020), and pyroptosis confers a decisive contribution to vascular endothelial dysfunction during the development of related diseases. Previous studies have suggested that endothelial cell’s pyroptosis was associated with cardiovascular diseases, including atherosclerosis (Zhang L. et al., 2019) and hypertension (Wu et al., 2022). Besides, the activation of NLRP3, ASC, Caspase-1, and GSDMD is increased significantly in atherosclerotic endothelial cells (Zhang et al., 2018). Furthermore, NLRP3 inflammasome, Caspase-1, and IL-1β trigger inflammation in the blood vessel wall, thereby leading to atherosclerosis (Karasawa and Takahashi 2017). Oxidized low-density lipoprotein (ox-LDL) and cholesterol crystals are abundant in atherosclerotic lesions (Zhang et al., 2015; Keping et al., 2020). Ox-LDL (Keping et al., 2020) and cholesterol crystals (Duewell et al., 2010; Zhang et al., 2015) could also promote NLRP3 inflammasome and Caspase-1 activation, leading to the release of IL-1β and IL-18 in immune cells. Especially, NLRP3 inflammasome promotes plaque formation and contributes to the development of atherosclerosis by affecting several targets, including signal transducer and activator of transcription (STAT), mitogen-activated protein kinases (MAPK), c-Jun N-terminal kinase (JNK), microRNA network, reactive oxygen species (ROS), and protein kinase R (PKR) (Hoseini et al., 2018). Thus, endothelial cell’s pyroptosis contributes to atherosclerosis formation and development by accelerating the release of inflammatory cytokines and increasing the vascular permeability (Zhaolin et al., 2019).

Additionally, NLRP3-dependent pyroptosis mediates endothelial dysfunction, which provides an impetus for hypertension (Pasqua et al., 2018), cardiovascular complications of coronary heart disease, and atherosclerosis in endothelial cells. The study highlighted that pyroptosis is a significant mediator of vascular dysfunction and injury in hypertensive patients (De Miguel et al., 2021). The serum level of IL-1β was higher in patients with essential hypertension than in healthy persons (Zeng et al., 2019). Besides, the research shows that the downregulation of the expression of key components of the NLRP3 inflammasome can delay the development of hypertension (De Miguel et al., 2021). The study found that microcrystals, and high levels of extracellular ATP and ROS could activate the NLRP3 inflammasome in the hypertensive patients (Krishnan et al., 2014). Overall, the endothelial cell’s pyroptosis is closely associated with the development of cardiovascular diseases. Aerobic exercise is an ideal non-drug management to inhibit endothelial cell’s pyroptosis and takes an essential role in treating cardiovascular diseases.

### Effect of aerobic exercise on endothelial cell’s pyroptosis

Aerobic exercise is beneficial for maintaining the function of vascular endothelial cells (Kourek et al., 2021). Notably, aerobic exercise could significantly alleviate the endothelial dysfunction and reduce the risk of cardiovascular diseases (Neunhäuserer et al., 2021). The study also showed that aerobic exercise could increase the blood flow and laminar shear stress as well as reduce leukocyte adhesion (You et al., 2013), and risk of inflammation, thereby improving the antioxidant system of enzymes and immune responses. Many studies have found that endothelial cell’s pyroptosis can be inhibited by aerobic exercise (Lee et al., 2018; Lee et al., 2020). Lee et al. (2018) have proved that voluntary running could reduce the activation of NLRP3 inflammasome in the endothelial cells of the coronary arteries. Their findings further suggested that aerobic exercise improves the vascular function by inhibiting NLRP3 inflammasome signaling (Lee et al., 2020). Other studies also reported that treadmill exercise of >12 weeks could reduce the endothelial cell’s pyroptosis in arteriosclerosis (Hong et al., 2018; Hong et al., 2021) (as shown in Table 1).

**TABLE 1:** Effects of aerobic exercise on pyroptosis-related factors in different cells.

Cell types	Object	Diseases	Exercise pattens	Effect of exercise	References
Endothelial cells	Mouse	Atherosclerosis	Treadmill training (12 weeks)	eNOS↑, Caspase-1↓	Hong et al. (2018)
Aortic endothelial	Mouse	Obesity	Voluntary wheel running (12–14 weeks)	IL-1β↓, NLRP3↓, Caspase-1↓, Oxidative stress↓	Lee et al. (2020)
Endothelial cells	Mouse	Atherosclerosis	Treadmill training (12 weeks)	NADPH↓, TXNIP/NLRP3↓, Oxidative stress↓	Hong et al. (2021)
Adipose tissue	Human	T2DM	Calorie restriction and exercise (1 year)	IL-1β↓, NLRP3↓, Caspase-1↓, IL-18↓	Vandanmagsar et al. (2011)
Adipose tissue	Human	T2DM and coronary artery disease	Endurance training combined with resistance training (1 year)	IL-18 = , Caspase1 = , NLRP3 = , Circulating IL-18↓	Zaidi et al. (2019)
Adipose tissue	Mouse	Obesity	Treadmill training (10 weeks)	IL-1β↓, IL-18↓, TNF-α↓	Mardare et al. (2016)
Adipose tissue	Mouse	HFD rats	Treadmill training (8 weeks)	NLRP3↓, FGF2↓	ZhuGe et al. (2020)
Prefrontal cortex	Mouse	Depression	Swimming (4 weeks)	NLRP3↓, Leptin↑	Liu et al. (2015)
Hippocampus	Mouse	Depression like behavior rats	Treadmill training (4 weeks)	IL-1β↓, NLRP3↓, Caspase-1↓, IL-18↓, Body weight↓	Wang et al. (2016b)
Hippocampus	Mouse	HFD-induced obese rats	Treadmill training (8 weeks)	IL-1β↓, NLRP3↓, Nrf2/Ho-1↑, BDNF↑	Cai et al. (2016)
Hippocampus	Mouse	T2DM rats	Treadmill training (4 weeks)	IL-1β↓, NLRP3↓, PI3K/AKT/mTOR↑, AMPK/Sirt↑, NF-κB/NLRP3/IL-1β↓	Li et al. (2019)
Prefrontal cortex	Mouse	Diabetic Rats	Treadmill training (4 weeks)	NLRP3↓, PI3K/AKT↑, NF-κB↓	Wang et al. (2019)
Hippocampus	Mouse	Alzheimer disease	Treadmill training (12 weeks)	NLRP3↓, IL-1β↓, Caspase-1↓, ASC↓	Liang et al. (2020)
Hippocampus	Mouse	Post-stroke drepression	Treadmill training (4 weeks)	NLRP3↓, TLR4↓, NF-κB↓	Li et al. (2020)
Hippocampus	Mouse	Alzheimer disease	Treadmill training (4 weeks)	NLRP3↓, TXNIP↓, Caspase-1↓, ASC =	Rosa et al. (2021)
Brains	Mouse	Parkinson’s disease	Treadmill training (6 weeks)	IL-1β↓, NLRP3↓, Caspase-1↓, Oxidative stress↓, TLR4↓, NF-κB↓, ASC↓	Wang et al. (2021)
Neuronal tissue	Mouse	Hyperlipidemia	Swimming (12 weeks)	NLRP3↓, IL-18↓, Caspase-1↓	Bai et al. (2021)

Note: “↓” indicates that its expression can be downregulated by exercise, “↑” indicates that it can be enhanced by exercise, “=” indicates that the change is not obvious by exercise.

### Aerobic exercise reduces vascular inflammation

Accumulating evidence has demonstrated that NLRP3 inflammasome plays a vital role in vascular inflammation (Wang L. et al., 2016). In endothelial cells, NLRP3 inflammasomes could be activated in response to multiple stimuli and are involved in vascular pathology (Lee et al., 2020).

Stimuli, including oxidative stress, mitochondrial dysfunction and lysosomal rupture have been demonstrated to activate the NLRP3 inflammasomes (Hoseini et al., 2018), which are important initiators in the development of vascular diseases. Moreover, ox-LDL and cholesterol crystals stimulate nuclear factor-κB (NF-κB) activation and TNF-α secretion (Steyers and Miller 2014). Then, the activated NF-κB further affects the NLRP3 signaling and contributes to the development of atherosclerosis (Hoseini et al., 2018). Studies have demonstrated that 12 weeks of treadmill exercise could down-regulate NF-κB protein expression and inhibit NF-κB-mediated aortic inflammation in participants (Wu et al., 2017).

Additionally, NLRP3 inflammasomes can be activated by the thioredoxin-interacting protein (TXNIP), which plays a crucial role in inflammatory response (Byon et al., 2015). The TXNIP/NLRP3 inflammasome signaling is closely associated with the development and progression of atherosclerosis (Hoseini et al., 2018). The activated NLRP3 inflammasome could increase the expression and release of the high-mobility histone box-1 (HMGB1) in endothelial cells (Lee et al., 2020), promoting endothelial hyperpermeability and leading endothelial dysfunction (Wang L. et al., 2016; Wang et al., 2016a). Several studies have demonstrated that aerobic exercise can significantly reduce vascular inflammation by inhibiting NLRP3 inflammasome, HMGB1, and its downstream effects (Goh and Behringer 2018; Kar et al., 2019; Lee et al., 2020).

### Aerobic exercise improves endothelial cell function

Vascular elasticity is regulated by generating many potent vasoactive substances, including vasodilator nitric oxide (NO) and contractile factor endothelin-1 in endothelial cells (Haybar et al., 2019). NO is a vasomotor factor produced and released by vascular endothelial cells, which has an important protective effect on the vascular wall and endothelial function (Ferentinos et al., 2022). NO bioavailability refers to the production and utilization of NO in endothelial cells, which is closely related with endothelial dysfunction. The reduction of NO bioavailability reportedly resulted from oxidative stress and expression of inflammatory factors (Chen et al., 2018). Similarly, a previous study has found that NO inhibits NLRP3 activation, thereby preventing pyroptosis in endothelial cells (Jiang et al., 2020).

Aerobic exercise is a promising non-medical treatment for preventing early endothelial dysfunction and redox imbalance by increasing NO bioavailability and reducing chronic inflammation (Gao et al., 2021). Moreover, aerobic exercise can effectively increase the NO content and enhance the diastolic function of vascular endothelial cells (Gao et al., 2021). NO can further increase the blood flow in the body during aerobic exercise.

In summary, aerobic exercise could regulate NO production and bioavailability to improve the endothelial cell’s function. Firstly, aerobic exercise increased the NO bioavailability by enhancing phosphorylated eNOS expression and reversing aortic endothelial dysfunction. In the vascular endothelium, aerobic exercise improves the NO bioavailability by enhancing endothelial NO synthase (eNOS) expression and eNOS/NO signaling (Lee et al., 2018), decreasing oxidative stress and inflammatory pathways. Aerobic exercise can enhance the heart’s pumping function, increase the heart’s output, accelerate the blood flow and blood shear stress, thereby stimulating the NO synthesis by vascular endothelial cells (Inoue et al., 2020). Secondly, aerobic exercise could improve the NO production by aggrandizing adiponectin (APN) and AdipoR1 levels (Lee et al., 2020). Thirdly, aerobic exercise can elevate the expression of junction proteins zonula occludin-1 (ZO-1) and ZO-2 (these are associated with endothelial permeability and dysfunction (Wang L. et al., 2016)) in endothelial cells, thereby facilitating NO production (Lee et al., 2020). Lastly, aerobic exercise induces the activity of superoxide dismutase (SOD), which results in the decrease in ROS production and ultimately improves the generation of NO (Cao et al., 2020). Studies have indicated that aerobic exercise could improve the endothelial cell’s function by downregulating TXNIP/NLRP3 inflammasome signaling (Hong et al., 2021). Overall, aerobic exercise could inhibit endothelial cell’s pyroptosis by improving the vascular endothelial cell’s function.

### Aerobic exercise decreases oxidative stress

**FIGURE 2:**
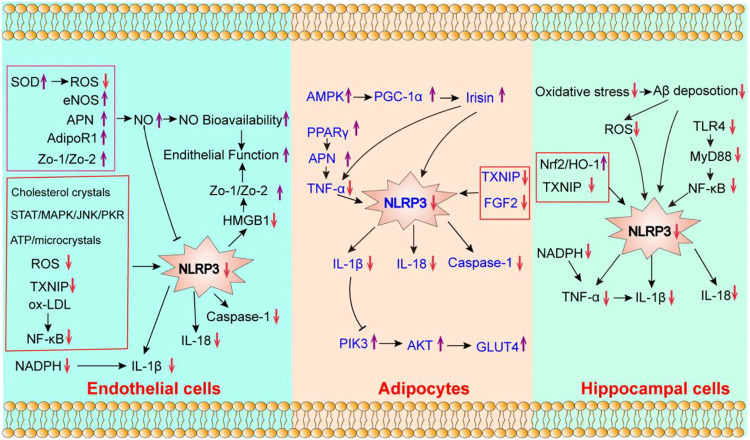
The potential effect of aerobic exercise on the pyroptosis of endothelial cells, adipocytes and hippocampal cells. “↓” indicates that its expression can be downregulated by aerobic exercise; “↑” indicates that it can be enhanced by aerobic exercise.

Nicotinamide adenine dinucleotide-phosphate (NADPH) oxidases take a vital role in oxidative stress. NADPH oxidases could produce superoxides (O^2−^), which induce reactive free radicals (Gjevestad et al., 2015) and act as the main source of ROS in blood vessels. The ROS-dependent activation of NLRP3 inflammasome can induce endothelial impairment (Rovira-Llopis et al., 2018) and oxidative stress. Previous studies have shown that NADPH subunit p22phox decreased the expression of IL-1β (Liao et al., 2020). Aerobic exercise could inhibit superoxide production and NADPH oxidases activity in coronary arteries (Hong et al., 2021), thereby reducing ROS production and oxidative stress (Cunha et al., 2017). Treadmill exercise reportedly could suppress ROS production by reducing the activity of NADPH oxidases (Jeong et al., 2018). As shown in Figure 2, the potential mechanisms of aerobic exercise modulating endothelial cell’s pyroptosis are as follows: 1) reduces vascular inflammation by inhibiting the expression of NLRP3 inflammasome. 2) improves endothelial function by enhancing NO bioavailability, and 3) decreases oxidative stress by reducing the activity of NADPH oxidase and IL-1β.

## Aerobic exercise and adipocyte pyroptosis-related diseases

Adipocytes are closely related to metabolism, and aerobic exercise plays an important role in improving metabolic diseases by regulating adipocyte’s pyroptosis. Studies have shown that targeting the NLRP3 inflammasome would reduce diet-induced metabolic abnormalities in mice (Chiazza et al., 2016; Ding et al., 2019).

### Adipocyte’s pyroptosis and its related diseases

Adipose tissue is the largest endocrine organ of the human body capable of storing lipids, secreting a large amount of adipokines, and it takes an essential role in the metabolism of human nutrients (Carbone et al., 2019). Chronic inflammation and adipose tissue dysfunction usually occur in individuals or mice with diabetes or obesity (Šimják et al., 2018). Adipocyte’s pyroptosis is an important upstream event in metabolism-related diseases including obesity (Giordano et al., 2013) and diabetes (Vandanmagsar et al., 2011). The expressions of Caspase-1, NLRP3, and other related factors of adipocyte’s pyroptosis were abundantly present in obese patients and mice (Giordano et al., 2013), which are involved in systematic inflammation and glucose homeostasis of adipose tissues (Ding et al., 2019; Wu et al., 2020). Additionally, the elevated expressions of the NLRP3 inflammasome, IL-1β, and IL-18 in adipose tissues are directly associated with insulin resistance and severity of diabetes (Esser et al., 2013). Mitochondria are reportedly involved in regulating NLRP3 inflammasome activation in adipocytes (Zhang et al., 2021). Moreover, a previous study found that high-fat diet induced overactivation of NLRP3 inflammasome in mice, the protein expression of genes related to mitochondrial biogenesis decreased, suggesting that mitochondrial damage caused by glucose and lipid metabolism disorders may activate the NLRP3 inflammasomes (Zhang et al., 2021). Therefore, the adipocyte’s pyroptosis is mainly related to metabolic diseases. Further, aerobic exercise is an effective strategy to prevent metabolic diseases by limiting the adipocyte’s pyroptosis.

### The potential mechanism of aerobic exercise on adipocyte’s pyroptosis

Lipids have important biological functions, in fact, fat is the energy provider in our body. The prominent roles of adipose tissue are to sequester fatty acids in times of energy excess and to release fatty acids via the process of lipolysis during times of high-energy demand, such as during an exercise. (Tsiloulis and Watt 2015). Several studies demonstrated that aerobic exercise could improve the function of adipocytes (Stanford et al., 2015), alter the expression of adipokines (Stinkens et al., 2018), and decrease adipocyte’s inflammation. Aerobic exercise training has been reported to inhibit the expression of pro-inflammatory factors in adipocytes, promotes the balance of the oxidative and antioxidant systems, and improves the inflammatory state. Researches have also demonstrated that 10 weeks of aerobic exercise ameliorates HFD-induced complications through the reduction of NLRP3, IL-18, TNF-α, TLR4 and IL-1β activation in adipocytes (Mardare et al., 2016). Therefore, aerobic exercise training is an effective strategy to reduce the expression of pyroptosis-related factors in adipocytes. As shown in Table 1, previous studies have shown that treadmill exercise training for >8 weeks can decrease the release of pyroptosis-associated factors in the adipocytes of obese or HFD rats (Mardare et al., 2016; ZhuGe et al., 2020). Nevertheless, the molecular mechanism of the effect of aerobic exercise on adipocyte’s pyroptosis remains unclear.

### Aerobic exercise reduces adipocyte inflammation

Inflammation in adipocytes plays a vital role in metabolic diseases, as it increases the expression of NLRP3 and its related inflammatory factors (Vandanmagsar et al., 2011). TXNIP (Wu et al., 2020) and FGF2 (ZhuGe et al., 2020) can exacerbate the inflammatory response in adipocytes by activating NLRP3 inflammasomes and Caspase-1. A previous study has shown that 8 weeks of treadmill training effectively inhibited the NLRP3 expression and reduced the FGF2 levels in adipose tissues (ZhuGe et al., 2020). Moreover, it has been reported that TNF-α is responsible for regulating the transcription of NLRP3 inflammasome components and inflammatory molecules in cryopyrinopathies (McGeough et al., 2017). Similar studies confirmed that the expression of NLRP3 was positively correlated with the release of TNF-α in adipose tissues (Bauernfeind et al., 2016). The increase in peroxisome proliferator-activated receptor-γ (PPARγ) levels raises the expression of APN as well as inhibits TNF-α release (Xia 2015). As it is known, PPARγ is responsible for regulating adipocyte differentiation (Ahmadian et al., 2013). In adipose tissues, the expression levels of PPARγ and APN could be increased significantly after aerobic exercise, while that of TNF-α was decreased (Xia 2015). Moreover, after 10 weeks of treadmill training, the significantly decreased expressions of NLRP3, TNF-α, and IL-1β were observed in adipose tissues (Mardare et al., 2016). The above mentioned results suggest that aerobic exercise training may inhibit adipocyte’s pyroptosis by reducing adipocyte inflammation.

Irisin, also known as fibronectin domain-containing protein 5 (FNDC5), is an exercise-inducing factor; it is not only a muscle factor but also an adipocytokine. A previous study has shown that irisin is a promising therapeutic agent that inhibits NLRP3-mediated pyroptosis of cardiomyocytes (Yue et al., 2021). AMP-activated protein kinase (AMPK) is essential for maintaining peroxisome proliferator-activated receptor-coactivator-1α (PGC-1α) (Gholamnezhad et al., 2020) and irisin (Lally et al., 2015) expressions. Irisin could inhibit the ROS/NLRP3 inflammatory signaling (Peng et al., 2017), TNF-α (Clark and Vissel 2019), and pyroptosis (Yue et al., 2021). Aerobic exercise has been demonstrated to activate AMPK and PGC1-α (Lally et al., 2015), increasing irisin expression in adipose tissues (Sanchez-Delgado et al., 2015) and inhibiting the NLRP3 related signaling.

### Aerobic exercise ameliorates insulin resistance

Phosphatidylinositol 3-hydroxy kinase (PI3K)/protein kinase B (AKT) signaling has been regarded as a key signaling pathway in glucose homeostasis, lipid metabolism and insulin resistance (Abeyrathna and Su 2015). The activation of the NLRP3 inflammasome could enhance the expression of IL-1β, IL-18, and interferonγ (IFNγ), while it inhibits IRS-1/PI3K/AKT signaling (Sun et al., 2017) (Vandanmagsar et al., 2011), thereby leading to insulin resistance. Vandanmagsar et al. (2011) proved that aerobic exercise ameliorated insulin resistance in the adipose tissues of T2DM patients by inhibiting the expression of NLRP3 and IL-1β. Another study found that the decreased expression of IL-1β and NLRP3 was positively associated with decreased blood glucose levels and improved insulin resistance index (Vandanmagsar et al., 2011). Moreover, aerobic exercise could enhance the expression of PI3K and AKT, and sequentially activate the PI3K/AKT/glucose transporter 4 (GLUT4) signaling pathway in adipose tissues, thereby improving insulin sensitivity (Yi et al., 2020). Taken together, ameliorating adipocyte’s inflammation and insulin resistance are the potential molecular mechanisms of the effects of aerobic exercise on adipocyte’s pyroptosis (Figure 2).

## Aerobic exercise and hippocampal cell pyroptosis-related diseases

Hippocampal cell’s pyroptosis is closely related to the development of neurodegenerative diseases (Han et al., 2020; Li et al., 2021), and aerobic exercise is an ideal regimen to inhibit pyroptosis of hippocampal cells, which is beneficial for patients with neurodegenerative diseases.

### Hippocampal cell’s pyroptosis and its related diseases

The hippocampal cells take a vital role in storing information associated with memory. Pyroptosis of hippocampal cells is closely associated with AD’s pathogenesis (Han et al., 2020), depression (Li et al., 2021), and so on. Neuroinflammation mediated by hippocampal cells and microglia take a crucial role in AD, primarily owning to amyloid-beta (Aβ) deposition and pyroptosis. The inhibition of NLRP3 in AD mice reduced Caspase-1 expression and Aβ deposition, and improved the cognitive function (Dempsey et al., 2017). Moreover, the activation of IL-1β and GSDMD will induce neuronal pyroptosis, and plays a significant role in the pathogenesis of AD (White et al., 2017; Han et al., 2020).

Current evidence has demonstrated that the NLRP3-mediated pyroptosis was a key modulator in the development of depression (Li et al., 2021). Especially, the NLRP3 inflammasome promotes hippocampal neurons and depression-like behavior in the hippocampus in depressed rats (Herman and Pasinetti 2018; Yang et al., 2020).

In fact, the downstream cytokines of NLRP3, including IL-1β and TNF-α, were increased in the cerebral spinal fluid and serum of patients with depression (Herman and Pasinetti 2018). In brief, the hippocampal cell’s pyroptosis is closely related to AD and depression. Aerobic exercise is an important way to suppress hippocampal cell’s pyroptosis in patients with neurodegenerative diseases.

### The molecular mechanism of the effect of aerobic exercise on hippocampal cell’s pyroptosis

Emerging evidence indicates that aerobic exercise can improve the function of hippocampal cells (Zhang X. et al., 2019). The possible mechanism is that aerobic exercise effectively reduces Aβ deposition by regulating neuroinflammation and oxidative stress (Zhang X. et al., 2019). Some studies have proved that aerobic exercise can inhibit hippocampal cell’s pyroptosis. As shown in Table 1, the studies indicated that aerobic exercise could inhibit NLRP3 inflammasome-related inflammatory cytokines, including Toll-like receptor 4 (TLR4), NF-κB, TXNIP, IL-1β, and IL-18. As mentioned above, these studies have suggested that aerobic exercise can reduce the expression of pyroptosis-related factors in the hippocampal cells. Aerobic exercise could inhibit the TLR4/NF-κB/NLRP3 signaling pathway in the dentate gyrus region of the hippocampus of post-stroke depression models (Li et al., 2020), which could prevent the activation of TXNIP and NLRP3 inflammasome pathways in AD rats (Rosa et al., 2021), and ameliorate depression-like behaviors by decreasing NLRP3, IL-1β, and IL-18 expressions in the hippocampal tissues (Wang et al., 2016c). These studies suggested that aerobic exercise could reduce hippocampal cell’s pyroptosis.

### Aerobic exercise reduces microglia activation

Microglia are the major source of inflammatory cytokines in the central nervous system (Habib and Beyer 2015) and coordinate the brain’s inflammatory response (Andoh and Koyama 2020). Studies have demonstrated that TLR4 could activate microglia, which transmit downstream inflammatory signals through the adaptor protein MyD88 (Kang et al., 2016), then activate NF-κB and NLRP3 inflammasome. NLRP3 inflammasome has been demonstrated to activate the microglia (Freeman et al., 2017), and NLRP3 protein was preferentially expressed in the microglia (Xia et al., 2021). The NLRP3 complex secretes IL-1β and IL-18, leading to pro-inflammatory response and pyroptosis (Zhou et al., 2011).

Numerous studies have found that aerobic exercise upregulated the expression of anti-inflammatory cytokines, thereby inhibiting the activation of microglia (Andoh and Koyama 2020) and expression of the NLRP3 inflammasome (Wang et al., 2016b). Aerobic exercise can inhibit microglial activation by decreasing the levels of IL-1β and TNF-α (Zhang X. et al., 2019), and regulating TLR signaling pathways (Mee-Inta et al., 2019). Long-term treadmill running could also reduce the expression of IL-1β and IL-18, inhibiting microglial activation caused by the activation of NLRP3 inflammasome in the hippocampal tissues (Wang et al., 2016b). Therefore, aerobic exercise can inhibit hippocampal cell’s pyroptosis by reducing microglial activation.

### Aerobic exercise protects neurons by decreasing neuroinflammation

Neuroinflammation is an immune response mediated by cytokines released from the microglia, which is related to increased expression of inflammatory cytokines, including NLRP3, IL-1β, and IL-18, in the hippocampal cells. Aerobic exercise has been shown to relieve neuroinflammation and protect neurons by decreasing the expression of NLRP3, IL-1β, and IL-18 (Wang et al., 2016b; Rosa et al., 2021). Wang et al. (2019) have shown that 4 weeks of treadmill exercise training inhibited neuroinflammation and played a neuroprotective role by suppressing the NF-κB/NLRP3 signaling pathway. The potential mechanism for aerobic exercise inhibits the expression of hippocampal NLRP3 inflammasome by reducing the TXNIP levels in the hippocampal dissection (Rosa et al., 2021). Moreover, TXNIP mediates the activation of NLRP3-related inflammatory signaling pathways through oxidative stress (Italiani et al., 2018). Moreover, aerobic exercise activates the Nrf2/HO-1 pathways, although it suppresses the NLRP3/IL-1β pathway (Cai et al., 2016), thereby inhibiting hippocampal cell’s pyroptosis.

Multiple studies have demonstrated that aerobic exercise could inhibit upstream signaling of hippocampal cell’s pyroptosis. Specifically, Qu et al. have demonstrated that 8 weeks of aerobic exercise training inhibited the TLR4/myeloid differentiation 88 (MyD88)/NF-κB signaling pathway in the hippocampal tissue (Qu et al., 2020). Li et al. (2020) found that 28 days of running training inhibited the TLR4/NF-κB/NLRP3 inflammatory signaling pathway, which mediates the hippocampal neurons’ protective effect in post-stroke depressed mice. Qu et al. (2019) identified that 8 weeks of moderate-intensity treadmill exercise significantly reduced the expression of TLR4 in the hippocampal tissue of mice, and activated the TLR4/miR-223/NLRP3 pathway axis, thereby improving the hippocampal function and promoting the repair of the damaged hippocampal tissue. Furthermore, Li et al. (2019) proved that 4 weeks of treadmill exercise could modulate the NF-κB/NLRP3/IL-1β signaling pathways in the hippocampal proteins. Moreover, long-term running wheel exercise training inhibited the expression of NADPH oxidase, and release of TNF-α and IL-1β, and induced the antioxidant and protective effects of microglia on nerves (Simioni et al., 2018). In other words, aerobic exercise could inhibit hippocampal cell’s pyroptosis by reducing neuroinflammation.

### Aerobic exercise decreases Aβ deposition

Aβ deposition is neurotoxic and can destroy the neurons, resulting in abnormal autophagy, blocking the clearance of Aβ, and affecting the cognitive function of neurodegenerative diseases. Aβ deposition promotes ROS production oxidative stress (Matěj et al., 2015) and activates the NLRP3 inflammasome in microglial cells *in vitro* and *in vivo* (Luciunaite et al., 2020). Aerobic exercise reduces microglia-mediated neuroinflammation, oxidative stress and Aβ deposition by inhibiting NLRP3 expression in the microglia (Zhang X. et al., 2019; Liang et al., 2020; Nakanishi et al., 2021). Together, these studies suggested that aerobic exercise could inhibit hippocampal cell’s pyroptosis by decreasing Aβ deposition.

## Summary and prospect

In summary, the review highlighted the close association between aerobic exercise and pyroptosis-related diseases, suggesting that aerobic exercise can alleviate the pyroptosis by regulating the NLRP3 inflammation signaling. Aerobic exercise inhibits endothelial cell’s pyroptosis by improving the endothelial function, while reducing vascular inflammation and oxidative stress. Moreover, aerobic exercise affects adipocyte’s pyroptosis by ameliorating adipocyte inflammation and insulin resistance. The potential mechanism of the effects of aerobic exercise on hippocampal cell’s pyroptosis is the reduction of microglial activation, neuroinflammation and Aβ deposition.

Different patterns of exercise have varying effects on cell pyroptosis. For example, Khakroo Abkenar et al. (2019) and Comassi et al. (2018) have found that one-time acute and acute high-intensity exercises can promote the activation of pyroptosis-associated protein, which are related to exercise intensity. However, aerobic exercise, resistance training and chronic high-intensity intermittent exercise can inhibit the activation of pyroptosis. Thus, further studies are needed to define the optimal effects of different patterns of exercise on specific cell’s pyroptosis and their molecular mechanism. Additionally, at present, animal experiments to investigate the effect of exercise on cell pyroptosis are more frequently performed, as compared to human experiments, which are scarce and more likely involved a small sample size. More in-depth research on the human body can provide a more scientific basis on the efficacy of exercise in regulating cell pyroptosis and promoting health. Therefore, more methodological, high-quality, and large-sized human studies are needed to determine the ideal patterns of exercise.
